# OTEH-2. High-dimensional analysis of spatial immune cell heterogeneity in glioblastoma reveals differences between contrast-enhancing and non-contrast-enhancing tumor rims

**DOI:** 10.1093/noajnl/vdab070.041

**Published:** 2021-07-05

**Authors:** Dionysios C Watson, Defne Bayik, Matthew Grabowski, Manmeet Ahluwalia, Alireza Mohammadi, Justin D Lathia

**Affiliations:** 1 Cleveland Clinic, Cleveland, OH, USA; 2 University Hospitals Cleveland Medical Center, Cleveland, OH, USA; 3 Miami Cancer Institute, Miami, FL, USA

## Abstract

**Background:**

Glioblastoma (GBM) is the most common primary malignant brain tumor in adults. GBM remains an incurable disease, with a median survival ~20 months. Complex intercellular interactions within the tumor microenvironment and spatial heterogeneity have challenged and impeded therapeutic efficacy. The non-contrast-enhancing (by T1-weighted MRI) rim of GBM is not always safely resectable and represents a major source of recurrence. We hypothesized that differential immune infiltration is an underlying factor of spatial heterogeneity in GBM, particularly in the non-contrast-enhancing tumor rim.

**Methods:**

Five patients with newly diagnosed GBM (ages 53–84) were recruited to a device feasibility study (NCT04545177) utilizing an intraoperative high-resolution MRI-based navigation system coupled with the NICO Myriad (a non-ablative semi-automated resection tool) and a coupled automated biological Tissue Preservation System (NICO APS) to sample spatially mapped regions of tumors in a reproducible and minimally destructive manner. We obtained brain tumor tissue from: (a) tumor core, (b) contrast-enhancing tumor rim and (c) non-contrast-enhancing tumor rim. Downstream processing consisted of digestion of tumor tissue (Miltenyi human tumor digestion kit) for subsequent single-cell isolation, viability assessment and immediate staining for multiparametric flow cytometry for immune profiling.

**Results:**

Viability varied across sampled regions (median 85%, range 52–100%). With the exception of 1 sample, viability was >70% in all specimens. High-dimensional analysis with 26 marker flow cytometry revealed spatial heterogeneity in the frequency of myeloid-derived suppressor cell subsets, regulatory T cells, CD8+ T cells, as well as expression of T cell activation and exhaustion markers.

**Conclusions:**

Semi-automated, spatially mapped intraoperative sampling of GBM with high viability of specimens is feasible and reproducible with the NICO Myriad and APS devices. High-dimensional analysis of immune cells in the GBM microenvironment captured the spatial heterogeneity of GBM. Future studies will expand on these observations by analyzing more patient specimens in combination with multiple omics assays.

